# An Updated Meta-Analysis Based on the Preclinical Evidence of Mechanism of Aconitine-Induced Cardiotoxicity

**DOI:** 10.3389/fphar.2022.900842

**Published:** 2022-06-08

**Authors:** Hong Jiang, Yating Zhang, Yi Zhang, Xiaobo Wang, Xianli Meng

**Affiliations:** ^1^ School of Pharmacy, and Research Institute of Integrated TCM and Western Medicine, Chengdu University of Traditional Chinese Medicine, Chengdu, China; ^2^ State Key Laboratory of Southwestern Chinese Medicine Resources, Innovative Institute of Chinese Medicine and Pharmacy, Chengdu University of Traditional Chinese Medicine, Chengdu, China; ^3^ Ethnic Medicine Academic Heritage Innovation Research Center, Chengdu University of Traditional Chinese Medicine, Chengdu, China

**Keywords:** aconitine, cardiotoxicity, preclinical evidence, ion channels, mitochondrial damage, NLRP3

## Abstract

**Background:** Most *Aconitum* species in traditional Chinese medicine have the effect of dispelling wind, dehumidifying, warming the meridian, and relieving pain. Aconitine is the characteristic chemical component with the function of anti-inflammation, analgesic, and heart-strengthening effects. However, improper use will produce cardiotoxicity and neurotoxicity. Currently, the mechanisms of cardiotoxicity caused by aconitine are wheels within wheels without being fully disclosed. The systematic review and meta-analysis were therefore conducted to summarize the available evidence of myocardial toxicity caused by aconitine.

**Methods:** We searched PubMed, Embase, Web of Science, National Knowledge Infrastructure, WANFANG, and VIP information database for relevant preclinical studies. All the data were analyzed by RevMan version 5.3.

**Results:** Thirty-two studies met the final inclusion criteria, including both *in vivo* and *in vitro* study types. After aconitine treatment, the heart rate of animals was obviously abnormal, and the morphology and function of myocardial cells were significantly changed. Aconitine can induce changes in the electrophysiological activity of cardiac myocytes by regulating Na^+^, Ca^2+^, and K^+^ currents. Meanwhile, the mechanisms of cardiotoxicity of aconitine may be related to triggering mitochondrial dysfunction by inducing mitochondrial apoptosis and autophagy. It should not be ignored that the overactivation of NLRP3 inflammasome also exacerbates aconitine’s cardiotoxicity.

**Conclusion:** The altered ion channels and mitochondrial function, as well as the signaling pathways interacting with NLRP3, may deserve further study for aconitine-induced cardiotoxicity.

## Introduction

**FIGURE 1:**
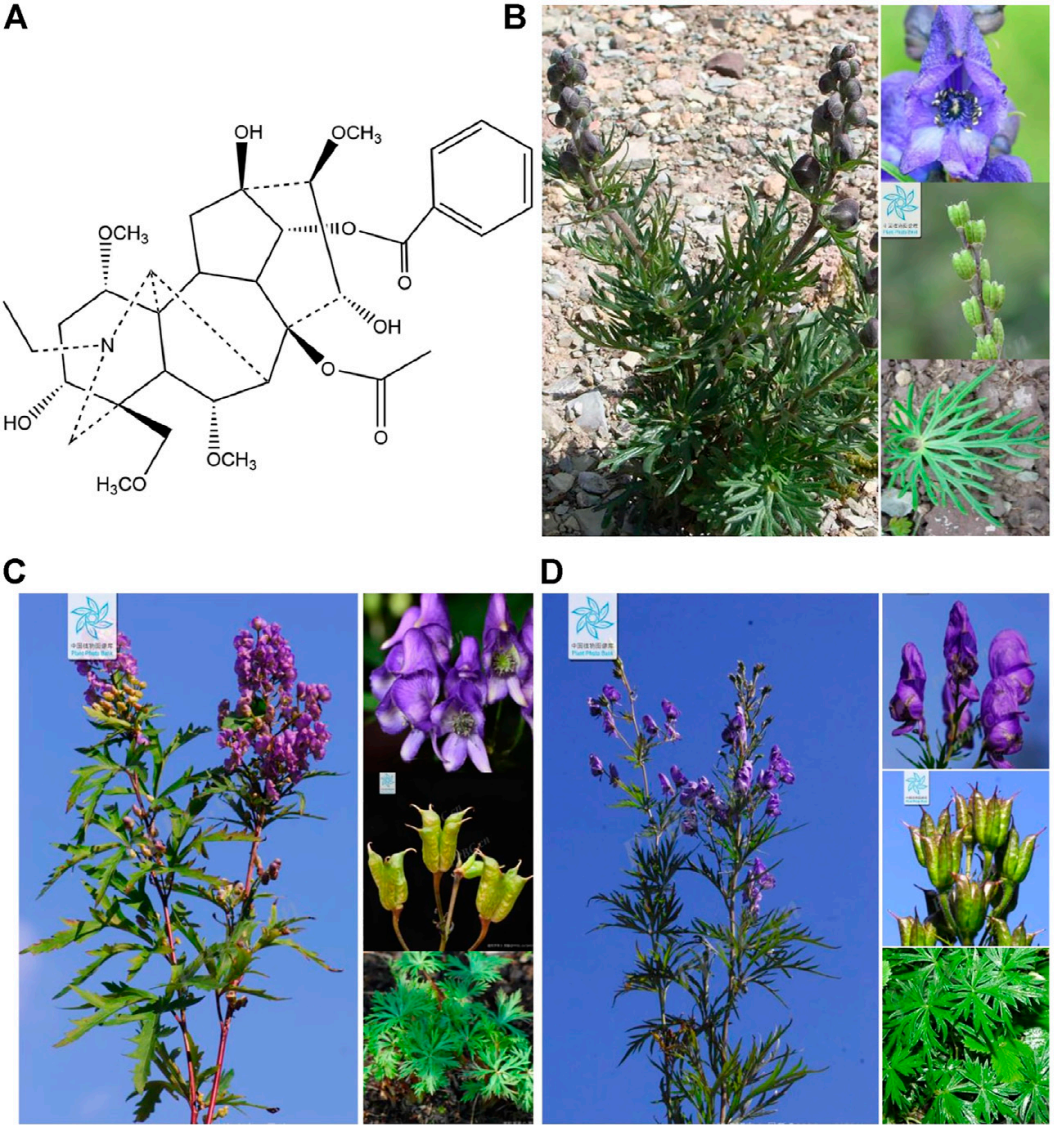
**(A)** Chemical structural formula of aconitine, **(B)** *Aconitum flavum* Hand.-Mazz., **(C)** *Aconitum kusnezoffii* Rchb., and **(D)** *Aconitum tschangbaischanense* S. H. Li et Y. H. Huang. The representative photographs of three *Aconitum* species were obtained from the Chinese flora online website (http://ppbc.iplant.cn/).

As common clinical traditional Chinese medicine in China, the main pharmacological effects of *Aconitum* species are cardiotonic, antihypertensive, anti-inflammatory, analgesic, and anti-tumor (Zhou et al., 2015), is highly regarded by physicians and pharmacists of various dynasties. According to historical documents and modern clinical reports, there are more than 600 prescriptions containing *Aconitum* species (Singhuber et al., 2009). Aconitine is a C_19_-diester diterpenoid alkaloid extracted from *Aconitum* species (Zhou et al., 2021), for example, *Aconitum flavum* Hand.-Mazz., *Aconitum kusnezoffii* Rchb., and *Aconitum tschangbaischanense* S. H. Li et Y. H. Huang (Figure 1). As the key substance that produces pharmacologic and toxicological effects, it has been widely reported that improper use of aconitine will cause toxicity to the cardiovascular system and central nervous system (Chan et al., 1993). By reviewing the previous literature, a total of 5,000 cases of aconitine poisoning were announced worldwide between 2001 and 2010, among which adverse cardiac events were the most serious clinical features (Li et al., 2016; Zhou et al., 2021). Therefore, the heart is the key target organ of aconitine toxicity. As reported, polymorphic ventricular arrhythmias, including the induction of ventricular premature beats (VPBs), atrioventricular blockade (AVB), ventricular tachycardia (VT), and ventricular fibrillation (VF), are the most common cardiac toxic side effects of aconitine (Lin et al., 2004; Ye et al., 2021; Zhou et al., 2021). Unfortunately, there is no effective antidote for aconitine-induced cardiotoxic events, which severely restricts the clinical use of aconitine-related traditional Chinese medicine or ethnic medicine. At present, the mechanism of aconitine-induced cardiotoxicity is not completely clear and needs further study. Hence, a comprehensive understanding of the molecular mechanism of aconitine cardiotoxicity will contribute to expanding the scope of clinical use of *Aconitum* medicinal materials.

In this study, the mechanism of aconitine induced cardiotoxicity was reviewed from the following aspects: 1) the interaction between ion channels leads to intracellular overload of Na^+^ and Ca^2+^ and induces arrhythmia (Ching et al., 2000; Zhou et al., 2005); 2) the mitochondrial damage leads to cellular energy metabolism disorder by balancing the redox signaling pathway during the process of energy metabolism (Sun et al., 2014); 3) the overexpression of apoptosis and autophagy-related proteins; 4) the activation of NLRP3 signaling pathway and its downstream caspase-1, IL-18, and IL-1β, leading to cardiac injury (Kang and Leaf, 1996; Yamamoto et al., 2000; Xiong et al., 2006; Gao et al., 2018). Meanwhile, we also give effective measures to reduce the cardiotoxicity of aconitine.

Systematic review is a powerful means of providing reliable information, which could be considered to be of the uppermost level of medical evidence. Based on the level of evidence from the Centre of Evidence-Based Medicine in Oxford, only data from a systematic review would be proposed as 1a-evidence (Glasziou et al., 2004). As an effective and widely accepted method, the review of a large amount of preclinical evidence of aconitine cardiotoxicity *in vivo* and *in vitro* will help to expand its clinical application and to deal with sudden poisoning events. Therefore, our goal is to determine the mechanism of aconitine cardiotoxicity by a comprehensive systematic review and meta-analysis.

## Methods

### Search Strategy

We used the public online databases of PubMed, Embase, Web of Science, National Knowledge Infrastructure (CNKI), WANFANG, and the VIP information to perform a comprehensive retrieval. Also then, we analyzed the *in vivo* and *in vitro* mechanism of aconitine-induced cardiotoxicity. From January 1980 to November 2021, all searches were documented electronically. The following was our technique for conducting a literature search: (aconitine OR aconite alkaloid OR aconitine alkaloids) AND (cardiotoxicity OR cardiac toxicity OR myocardial toxicity OR myocardial damage OR cardiovascular toxicity OR heart toxic OR heart toxicity).

### Study Selection

Experimental investigations evaluating the mechanisms of aconitine-induced cardiotoxicity were selected and incorporated. According to the above retrieval strategies, two authors were separately delegated to review the titles and/or abstracts, and then assess the qualifications of the full-text articles. The following criteria were used to determine whether or not a study should be included: 1) the article about aconitine causes cardiotoxicity; 2) the experimental group received aconitine monotherapy intervention despite mode, dosage, and frequency; 3) the major outcomes examined were the heart and myocardial cell injury (including at least one relevant indicator); 4) the control group received normal saline or no adjunct intervention. Correspondingly, the following were the predetermined exclusion criteria: 1) cardiotoxicity was not the target ailment; 2) aconitine was used in combination; 3) the article was a clinical study; 4) the study was a case report, clinical trial, review, abstract, comment, conference paper, or duplicate publication; and 5) no control group was included.

### Extraction of Information

The articles were evaluated by two independent reviewers, and the following particulars were retrieved from chosen studies: 1) the first author and the year of publication; 2) individual data from each study, such as animal species, gender, samples for individual comparison and weight; 3) type of anesthetic; 4) characteristics of intervention in treatment and control groups, such as drug, dosage, method of treatment, and frequency; 5) mean value, standard deviation, and inter-group difference of measurement and the corresponding data. If the presented data was incomplete and ambiguous, we attempted to contact the authors for more information or using digital ruler software else we just performed a qualitative analysis.

### Quality Evaluation

Two authors independently evaluated the methodological quality of the included studies based on the list of collaborative approach to meta-analysis and review of animal data from experimental studies (CAMARADES). The 10-item checklist was as follows (Sena et al., 2007): 1) peer-reviewed publication; 2) statements of temperature control; 3) randomly divided into treatment group or control group; 4) the model was induced by blind method; 5) blinded evaluation of results; 6) use of anesthetic had no apparent intrinsic myocardial preservation or neuroprotective effect; 7) use of animals with relevant comorbidities; 8) sample size calculation; 9) obedience with animal welfare regulations, and 10) declared any underlying conflict of benefits. Each research received an overall quality rating ground on a one-point system for each item. For any article we have questions about, we will immediately contact the corresponding author to discuss and negotiate.

### Statistical Analysis

RevMan version 5.3 software was employed to perform the pooled analyses. All of the outcome variables were treated as continuous data. The random effects model and standard mean difference (SMD) with 95% confidence intervals (CIs) were used to evaluate the mechanism of aconitine-induced cardiotoxicity. The *I* ^2^ statistics test was used to examine heterogeneity among individual research. If the probability value was <0.05, the difference was considered statistically significant.

## Results

### Study Inclusion

**FIGURE 2:**
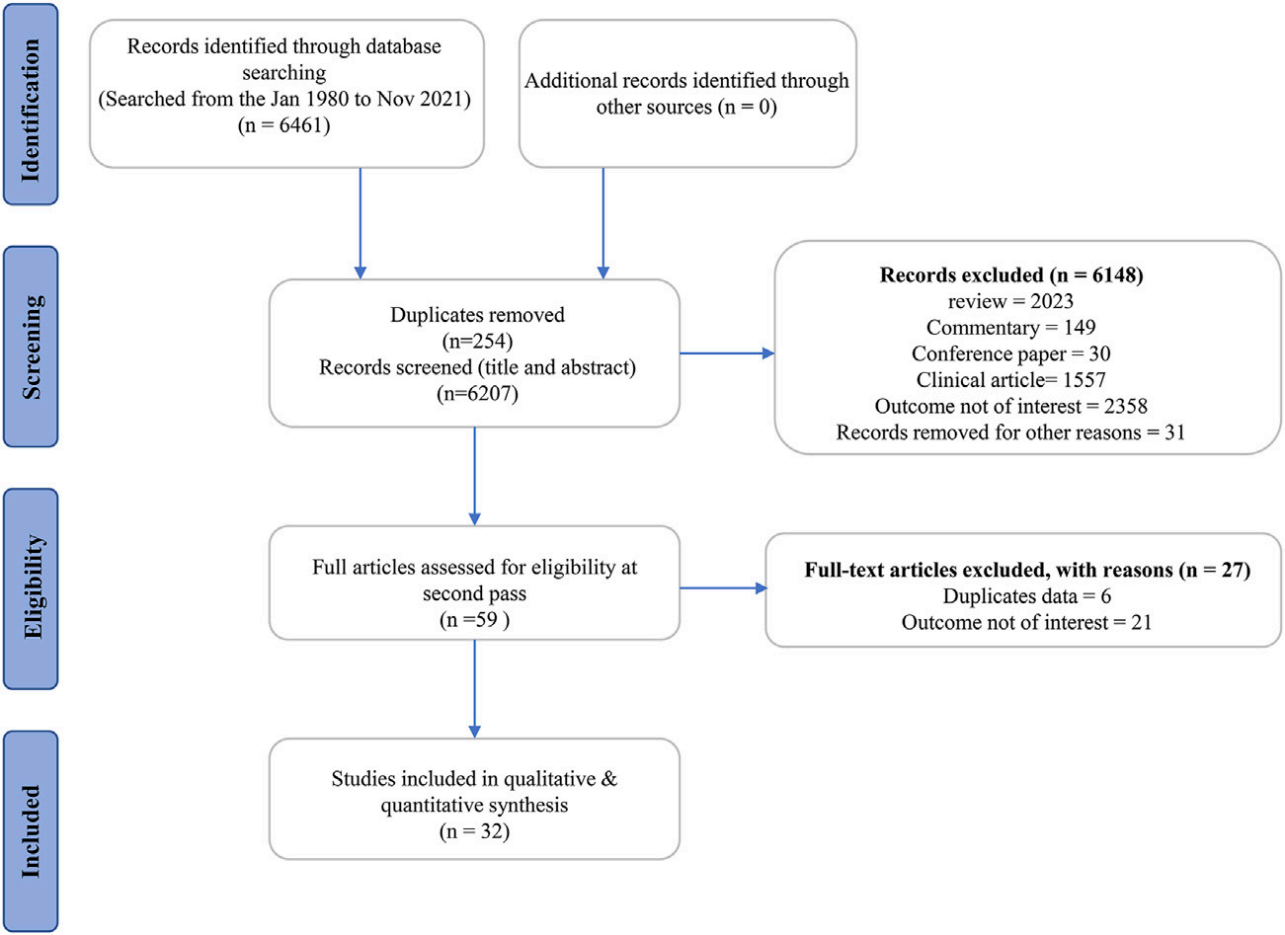
Flow chart of the database search and study identification.

Following a rigorous search of six databases, we found 6,461 publications with 6,207 records remaining after duplicates. Also, 6,148 publications were ruled out based on their titles and abstracts for at least one of the following reasons: 1) review article, commentary, conference article, or clinical study; 2) with no interesting outcomes; 3) other reasons, such as a letter to the editor and books. After scanning the complete text of the remaining 59 studies, six studies were eliminated because they are duplicate publications (such as dissertations and articles). Moreover, 21 studies were deleted because the outcome measurement was not of interest. Ultimately, 32 eligible articles (Liang et al., 1991; Meng, 2006; Fu, 2007; Liu, 2007; Wang, 2007; Wang et al., 2007; Zhang, 2007; Xu, 2008; Liu, 2009; Deng, 2010; Fang et al., 2012; Zhang et al., 2012; Wang, 2013; Zhou et al., 2013; Sun et al., 2014; Yu, 2015; Cui et al., 2018; Gao, 2018; Gao et al., 2018; Hu et al., 2018; Li et al., 2018; Zhang et al., 2018; Liu et al., 2019; Wang M. et al., 2020; Li et al., 2020; Peng et al., 2020; Zhang et al., 2020; Zhou et al., 2020; Xia et al., 2021; Yang et al., 2021; Ye et al., 2021; Wang S. et al., 2022) were identified for further evaluation, data extraction, and analysis (Figure 2).

### Characteristics of Included Studies

Table 1 summarized the essential characteristics of thirty-two qualifying research, between 1991 and 2021, with 12 studies done in English and 20 research conducted in Chinese. For study type and animal species, 27 studies were *in vitro* experiments, three studies were *in vivo* experiments, and two studies (Wang, 2013; Sun et al., 2014) had both *in vitro* and *in vivo* experiments. Of them, the animals used in three studies (Liang et al., 1991; Zhang et al., 2012; Sun et al., 2014) comprised Sprague–Dawley (SD) and Wistar rats, and two studies (Deng, 2010; Wang, 2013) guinea pigs. The weight of rats ranged from 150 to 350 g, and the weight of guinea pigs ranged from 300 to 350 g. Among which, zebrafish embryos were employed in seven studies (Fang et al., 2012; Cui et al., 2018; Liu et al., 2019; Wang S. et al., 2020; Li et al., 2020; Xia et al., 2021; Ye et al., 2021), H9c2 cell in nine studies (Gao, 2018; Gao et al., 2018; Hu et al., 2018; Zhang et al., 2018; Li et al., 2020; Peng et al., 2020; Zhang et al., 2020; Yang et al., 2021; Wang W. et al., 2022), primary cardiomyocytes of SD rats in 10 studies (Meng, 2006; Fu, 2007; Wang, 2007; Wang et al., 2007; Zhang, 2007; Xu, 2008; Liu, 2009; Zhou et al., 2013; Yu, 2015; Li et al., 2018), primary cardiomyocytes of Wistar rats in three studies (Liu, 2007; Sun et al., 2014; Zhou et al., 2020). Meanwhile, three studies chose primary cardiomyocytes of guinea pigs (Wang, 2013) and AC-16 cells (Cui et al., 2018; Zhou et al., 2020). For the choice of anesthesia in animal experiments, two studies (Zhang et al., 2012; Yu, 2015) used chloral hydrate, one study (Sun et al., 2014) ketamine/xylazine, and one study (Liu et al., 2019) MS-222, three studies (Wang, 2013; Zhou et al., 2013; Sun et al., 2014) pentobarbital, and the rest of studies was not reported. Of the 32 included studies, each one had different measured indicators, as well as different routes, times, and dosages of administration. In the results column, the arrow symbols pointing in different directions were used to indicate the trends of each indicator. The detailed information was summarized in Table 1.

**TABLE 1:** Basic characteristics of the included studies.

*In vivo* study							
**Study (year)**	**Species (gender)**	**Weight (g)**	**Anesthetic**		**Method of administration**	**Result**	**Intergroup difference**
Liang et al. (1991)	SD, male	150 ± 10	NR		0.5, 2, 4, 12, and 24 h after 1.46 mg/kg, i.g.	(1) ↓NADHD	(1) NR
Zhang et al. (2012)	SD, male	210–220	Chloral hydrate		0.2 ml/h, ivgtt	(1) ↑Heart rate (2) ↑RPP	(1) *p* < 0.05 (2) NR
Wang (2013)	Guinea pig, male	300–350	Pentobarbital sodium		25 μg/kg, IV	(1) Arrhythmia	(1) NR
Sun et al. (2014)	Wistar, male	220–240	Pentobarbital sodium		10 ml/kg for 3, 6, and 10 days, qd, i.g.	(1) Arrhythmia (2) ↑Apoptotic rate (3) Ultrastructural changes (4) Proteins expression: ↑RyR, ↑NCX, ↓Bcl-2, ↑P53, and ↑caspase-9/3	(1) NR (2) NR (3) NR (4) *p* < 0.05
Deng (2010)	Guinea pig, both	250–350	NR		3 × 10^−8^, 1 × 10^−7^, and 3 × 10^−7^ M	(1) ↓APD50/90, ↑VDD, and ↓APA	(1) *p* < 0.05
***In vitro* study**							
**Study (year)**	**Cell lines**			**Concentration**		**Result**	**Intergroup difference**
Zhang et al. (2020)	H9c2			10, 30, 50, 70, 90, and 110 μM		(1) ↓Cell viability (2) Protein expression: ↓α-actinin (3) Genes expression: ↓actc1, ↓myl1, ↓myl2, and ↓tnnt2	(1) *p* < 0.05 (2) NR (3) *p* < 0.01
Fang et al. (2012)	Zebrafish embryos			5, 10, 30, and 60 mg/L		(1) Pericardium edema and hemorrhage; ↑blood cells (2) ↑Heart rate	(1) NR (2) *p* < 0.05
Liu (2009)	Primary cardiomyocytes of SD neonatal rats			0.25, 0.5, 0.75, 1.0, 2.0, and 3.0 μg/L for 0.5, 1, 2, and 4 h		(1) ↓Pulse rhythm (2) ↑LDH (3) ↓Cell viability	(1) NR (2) *p* < 0.05 (3) *p* < 0.05
				0.1, 0.5, 1, and 2 μM for 0.5 h		(4) DNA damage: ↓HDNA%, ↓TDNA%, ↑TL, ↑TM, and ↑OTM	(4) *p* < 0.05
				1.0 μM for 1 h		(5) Proteins expression: ↓P-Cx43(Ser–368), ↑NP-Cx43(Ser–368), and ↓P-PKCα(Ser–657) (6) Protein expression: ↓P-PKCα (Ser–657) (7) Genes expression: ↑NCX, ↑RYR2, ↓SERCA2, and ↓PLB	(5) *p* < 0.01 (6) *p* < 0.01 (7) *p* < 0.01
Zhang (2007)	Primary cardiomyocytes of SD neonatal rats			0.25, 0.5, 0.75, 1.0, 1.5, and 2.0 μΜ for 1 h		(1) Proteins expression: ↓(P2+P1) Cx43 and ↑NP Cx43 (2) →Morphology (3) Proteins expression: ↓P-Cx43 (Ser–368) and ↑NP-Cx43 (Ser–368)	(1) *p* < 0.05 (2) NR (3) *p* < 0.01
Wang et al. (2007)	Primary cardiomyocytes of SD neonatal rats			100.0, 10.0, 1.0, and 0.1 μg/ml for 24 and 48 h		(1) ↓Cell viability (2) ↓MDA (3) ↑Beating rate (4) Cell shrinkage and ↑pseudopodia	(1) *p* < 0.01 (2) *p* < 0.01 (3) NR (4) NR
Meng (2006)	Primary cardiomyocytes of SD neonatal rats			3% for 0.5, 1 and 30 min		(1) Morphological changes (2) ↑LDH (3) ↑MDA (4) ↑Acid phosphatase activity (5) ↑Na+, ↓K+, ↑Ca+, and ↓Mg+ (6) ↓Na+-K+ATP (7) ↓OPTDM (8) ↓SDH (9) Cell cycle: ↑(G0/G1) %, ↓S% and ↓G2+M%	(1) NR (2) *p* < 0.05 (3) *p* < 0.05 (4) *p* < 0.05 (5) *p* < 0.05 (6) *p* < 0.01 (7) *p* < 0.05 (8) *p* < 0.05 (9) *p* < 0.01
Li et al. (2018)	Primary cardiomyocytes of SD neonatal rats			1, 5 and 10 μM		(1) ↓Ca^2+^ transient amplitude, ↑Ca^2+^ transient frequency (2) Enzymatic activities: ↓Ca^2+^-ATP, ↓Ca^2+^-Mg^2+^-ATP and ↓Na+-K+-ATP (3) ↑Na+, ↑Ca^2+^ and ↓K+ (4) Genes expression: ↓SERCA2 and ↑RyR2 (5) Proteins expression: ↓SERCA2 and ↑RyR2	(1) *p* < 0.01 (2) *p* < 0.05 (3) *p* < 0.05 (4) *p* < 0.05 (5) *p* < 0.05
Wang (2013)	Primary cardiomyocytes of guinea pig			Undefined multiple concentrations		(1) ↓APD30/90	(1) *p* < 0.01
Gao (2018)	H9c2			50, 100, 150, 200, and 250 μM		(1) ↓Cell viability	(1) *p* < 0.01
						(2) ↓Nuclear volume (3) ↑Apoptosis rate (4) ↑ROS (5) ↓Membrane potential (6) ↓ATP (7) Proteins expression: ↓PGC-1α and ↑Bax, ↑caspase-3, ↑cytochrome C, and ↓Bcl-2	(2) NR (3) *p* < 0.001 (4) *p* < 0.05 (5) *p* < 0.001 (6) *p* < 0.001 (7) *p* < 0.01
Cui et al. (2018)	Zebrafish embryos			1, 3, and 10 μM for 1 day		(1) Arrhythmia (2) ↓Stroke volume	(1) NR (2) *p* < 0.05
	AC-16			1 μM		(3) ↑Ca^2+^ oscillation frequency and ↓Ca^2+^ oscillation amplitude	(3) *p* < 0.05
Fu (2007)	Primary cardiomyocytes of SD neonatal rats			Undefined multiple concentrations		(1) ↓Pulse rhythm	(1) NR
				0.1, 0.25, 0.5, 1, and 3 μM for 0.5, 1, and 2 h		(2) ↓Cell viability (3) ↑LDH	(2) *p* < 0.05 (3) *p* < 0.05
				0.5, 1, and 3 μM		(4) DNA damage: ↑TL, ↓HDNA% and ↑TDNA%	(4) *p* < 0.05
				0.5 and 3 μM		(5) Calcium oscillation: ↓amplitude and ↑Ca^2+^	(5) *p* < 0.05
				0.5, 1, and 3 μM for 1 h		(6) Proteins expression: ↑RyR2 and ↑NCX (7) Genes expression: ↑RyR2 and ↑NCX	(6) *p* < 0.05 (7) *p* < 0.05
Wang (2007)	Primary cardiomyocytes of SD neonatal rats			3% for 1 min		(1) ↑α1-AR (2) →β1-AR (3) →β2-AR	(1) *p* < 0.05 (2) *p* > 0.05 (3) *p* > 0.05
				3% for undefined multiple time		(4) ↑Round and smaller cells and ↑contracted pseudopodia	(4) NR
				3% for 0.5, 1, and 30 min		(5) ↑IP3 (6) ↑cAMP/cGMP	(5) *p* < 0.05 (6) *p* < 0.01
Zhang et al. (2018)	H9c2			150, 250, 400, 500, and 1,000 μg/ml		(1) ↓Cell viability (2) ↓Cell volume	(1) *p* < 0.05 (2) NR
						(3) ↑LDH (4) ↑Apoptosis	(3) *p* < 0.05 (4) *p* < 0.05
Hu et al. (2018)	H9c2			0.05%, 0.1%, 0.2%, and 0.4% for 0.5, 1, 1.5, and 3 h		(1) ↓Cell viability	(1) *p* < 0.05
						(2) ↑Autophagy (3) ↑Apoptosis (4) Proteins expression: ↑LC3B, ↑beclin-1, ↑caspase-3, ↑Bcl-2, and ↑Bax	(2) NR (3) NR (4) *p* < 0.05
Li et al. (2020)	Zebrafish embryos			1.87, 3.75, 7.5, 15, and 30 μM for 48 h		(1) ↓Survival rate	(1) *p* < 0.05
				2 and 8 μM for 12, 24, 36, and 48 h		(2) ↓Contraction of ventricles and atria	(2) *p* < 0.05
						(3) ↑254 genes and ↓352 genes	(3) NR
	H9c2			Undefined multiple concentrations for 30 min		(4) ↓Survival rate (5) ↑Apoptosis	(4) *p* < 0.05 (5) *p* < 0.01
						(6) ↑Ca^2+^ (7) Proteins expression: ↓TnT, ↓Bcl-2, ↑caspase-3, and ↑Bax	(6) *p* < 0.01 (7) *p* < 0.05
Gao et al. (2018)	H9c2			0–250 μM for 24 h		(1) ↓Cell viability	(1) *p* < 0.01
				0, 100, and 200 μM for 24 h		(2) ↑Cell nuclei shrinkage and chromatin condensation (3) ↑Apoptosis (4) ↓ATP	(2) NR (3) *p* < 0.001 (4) *p* < 0.001
				0, 100, and 200 μM for 4 h		(5) ↑ROS (6) ↓Mitochondrial transmembrane potential (7) Proteins expression: ↓PGC-1α, ↓Bcl-2, ↑Bax, ↑caspase-3, and ↑cytochrome	(5) *p* < 0.05 (6) *p* < 0.001 (7) *p* < 0.01
Sun et al. (2014)	Primary ventricular myocytes of Wistar rats			1 μM		(1) ↑Beating rhythm (2) ↑Sarcomere shortening (3) ↑Ca^2+^ transient amplitude and ↑Ca^2+^	(1) NR (2) *p* < 0.01 (3) *p* < 0.01
				0.01, 0.04, 0.16, and 0.64 mΜ for 4 h		(4) ↓Cell viability (5) ↑LDH (6) ↑Apoptosis	(4) *p* < 0.01 (5) *p* < 0.05 (6) *p* < 0.01
Zhou et al. (2013)	Primary cardiomyocytes of SD neonatal rats			1 μM		(1) ↑Ca^2+^ (2) ↑APD50/90	(1) *p* < 0.05 (2) *p* < 0.01
				1 μM for 48 h		(3) Proteins expression: ↓SERCA2 and ↑NCX	(3) *p* < 0.05
Liu et al. (2019)	Zebrafish embryos			1, 2, and 4 μg/L for undefined multiple time		(1) Cardiac function: ↓Heart rate, ↓SV and ↓CO	(1) *p* < 0.05
				2.5 μg/L		(2) ↑Yolk sac hemorrhage and pericardial edema (3) ↑Apoptosis in the brain and eye area (4) Genes expression: ↓Nkx2.5, ↓Tbx5, and ↑caspase-3	(2) NR (3) NR (4) *p* < 0.05
Ye et al. (2021)	Zebrafish embryos			Undefined multiple concentrations for 2, 10, and 24 h		(1) ↑Heart rate (2) Curvature of the tail spine and enlarged pericardium	(1) *p* < 0.05 (2) NR
Peng et al. (2020)	H9c2, rat primary cardiomyocytes			Undefined multiple concentrations		(1) ↑503 genes and ↓314 genes (2) ↓Cell viability	(1) NR (2) *p* < 0.05
				1 μmol/L		(3) ↑ROS	(3) *p* < 0.05
				0.5 and 1 μM		(4) ↑Apoptosis (5) Proteins expression: ↑TNF-α and ↑FADD, ↑cytochrome C, ↑caspase-3/8, ↓Bcl-2, ↑RIP1/3, and ↑MLKL	(4) NR (5) *p* < 0.05
Wang et al. (2022a)	H9c2			3.125–400 μM for 24 h		(1) ↓Cell viability	(1) *p* < 0.01
				50, 100, and 200 μM for 24 h		(2) ↑LDH (3) Proteins expression: ↑beclin-1, ↑LC3, ↑p-AMPKα/AMPKα, and ↑p-ULK1/ULK1 (4) ↑ROS (5) ↑8-OHdG (6) ↓GSH	(2) *p* < 0.01 (3) *p* < 0.05 (4) *p* < 0.01 (5) *p* < 0.01 (6) *p* > 0.05
Zhou et al. (2020)	Primary ventricular myocytes of Wistar rats, AC-16 cells			0–320 μM for 24 h in AC-16 cells		(1) ↓Cell viability (2) ↑Mitochondrial superoxide productions (3) Notch1 signal is activated and then suppressed	(1) *p* < 0.05 (2) *p* < 0.05 (3) *p* < 0.05
				20 μM for 24 h in AC-16 cells		(4) Unchanged morphological characteristics and ultrastructure	(4) NR
				NRVMs 0–80 μM for 7 days in NRVMs		(5) →Cell viability (6) Unchanged morphological characteristics and ultrastructure (7) ↑Mitochondrial superoxide productions (8) Notch1 signal is activated and then suppressed	(5) NR (6) NR (7) *p* < 0.05 (8) *p* < 0.05
						(9) ↑Beating rate	(9) *p* < 0.05
Yang et al. (2021)	H9c2			0.25, 0,5, and 1.0 μM for 24 h		(1) ↑Apoptosis (2) ↑Ca^2+^ (3) Proteins expression: ↓Bcl-2, ↑Bax, ↑caspase-3, ↑p-p38 and ↑TRPV2 (4) Protein expression: ↑TRPV2	(1) *p* < 0.0001 (2) *p* < 0.01 (3) *p* < 0.001 (4) *p* < 0.05
Wang et al. (2020a)	Zebrafish embryos			15 mg/L for 48–72 h		(1) ↑Heart rate (2) ↑Pericardial edema, ↑blood cell, and ↑SV-BA distance (3) ↑696 genes and ↓925 genes	(1) *p* < 0.0001 (2) NR (3) NR
Yu (2015)	Primary cardiomyocytes of SD rats			0.3, 1, and 3 μM		(1) ↑Ca^2+^ oscillation amplitude and ↑Ca^2+^	(1) *p* < 0.05
Xu (2008)	Primary cardiomyocytes of SD neonatal rats			0.5, 1, and 3 μM for 15, 30, and 60 min		(1) ↓Cell viability	(1) NR
				0.5, 1, and 3 μM for 60 min		(2) DNA damage: ↑TDNA% and ↑TM	(2) *p* < 0.01
						(3) ↑ROS	(3) *p* < 0.01
Liu (2007)	Primary ventricular myocytes of Wistar rats			5, 20, 40, 80, 160, 320, 640, and 1,280 μM for 4, 8, and 12 h		(1) ↓Cell viability	(1) *p* > 0.05
				0.1, 0.3, and 0.6 mM for 24 h		(2) ↑LDH	(2) NR
				12.5, 25, 50, 100, and 200 μM		(3) Gene expression: ↓Kv4.3	(3) *p* < 0.05
Xia et al. (2021)	Zebrafish embryos			4, 6, 8, 10, 12, 14, 16, and 18 μM		(1) Yolk retention, swim-bladder deficiency, pericardial edema, and curved body shape (2) ↑Malformation rate, ↓body length and ↑SV-BA distance (3) ↑Heart rate	(1) NR (2) *p* < 0.05 (3) *p* < 0.01
				0.73, 2.42, 7.27, and 8.23 μM		(4) ↑ROS (5) ↓SOD (6) ↑MDA (7) ↑Apoptosis (8) Genes expression: ↑JNK, ↓Erk1/2, ↑Bax, ↑Bad, ↑cytochrome C, ↑caspase-3/9, and ↓Bcl-2	(4) *p* < 0.01 (5) *p* < 0.01 (6) *p* < 0.01 (7) *p* < 0.01 (8) *p* < 0.05

↑: demonstrates increasing trend; ↓: demonstrates decreasing trend; →: demonstrates the unchanging trend; APA, amplitude of action potential; APD, action potential duration; APD_30_, duration at 30% of repolarization; AR, adrenoceptor; cAMP/cGMP, cyclic adenosine monophosphate/cyclic guanosine monophosphate; CO, cardiac output; GSH, glutathione; HDNA%, head DNA%; IP3, inositol triphosphate; LDH, lactate dehydrogenase; MDA, malondialdenhyde; NADHD, reduce nicotinamide adenine dinucleotide dehydrogenase; NCX, Na^+^/Ca^2+^ exchanger; NR, not report; NRVMS, neonatal rat ventricular myocytes; OPTDM, average optical density of myocardial glycogen granules; OTM, olive tail moment; PLB, phospholamban; RPP, rate pressure product; ROS, reactive oxygen species; RyR_2_, ryanodine receptor; SERCA_2_, SR Ca^2+^-ATPase; SDH, succinate dehydrogenase; SD, Sprague–Dawley; SV, stroke volume; SV-BA, sinus venous and bulbus arteriosus; TDNA%, tail DNA%; TL, tail length; TM, tail moment; VDD, average velocity of diastolic spontaneous depolarization; 8-OHdG, 8-hydroxydeoxyguanosine.

### Cardiotoxic Mechanisms

#### Cell Viability

**FIGURE 3:**
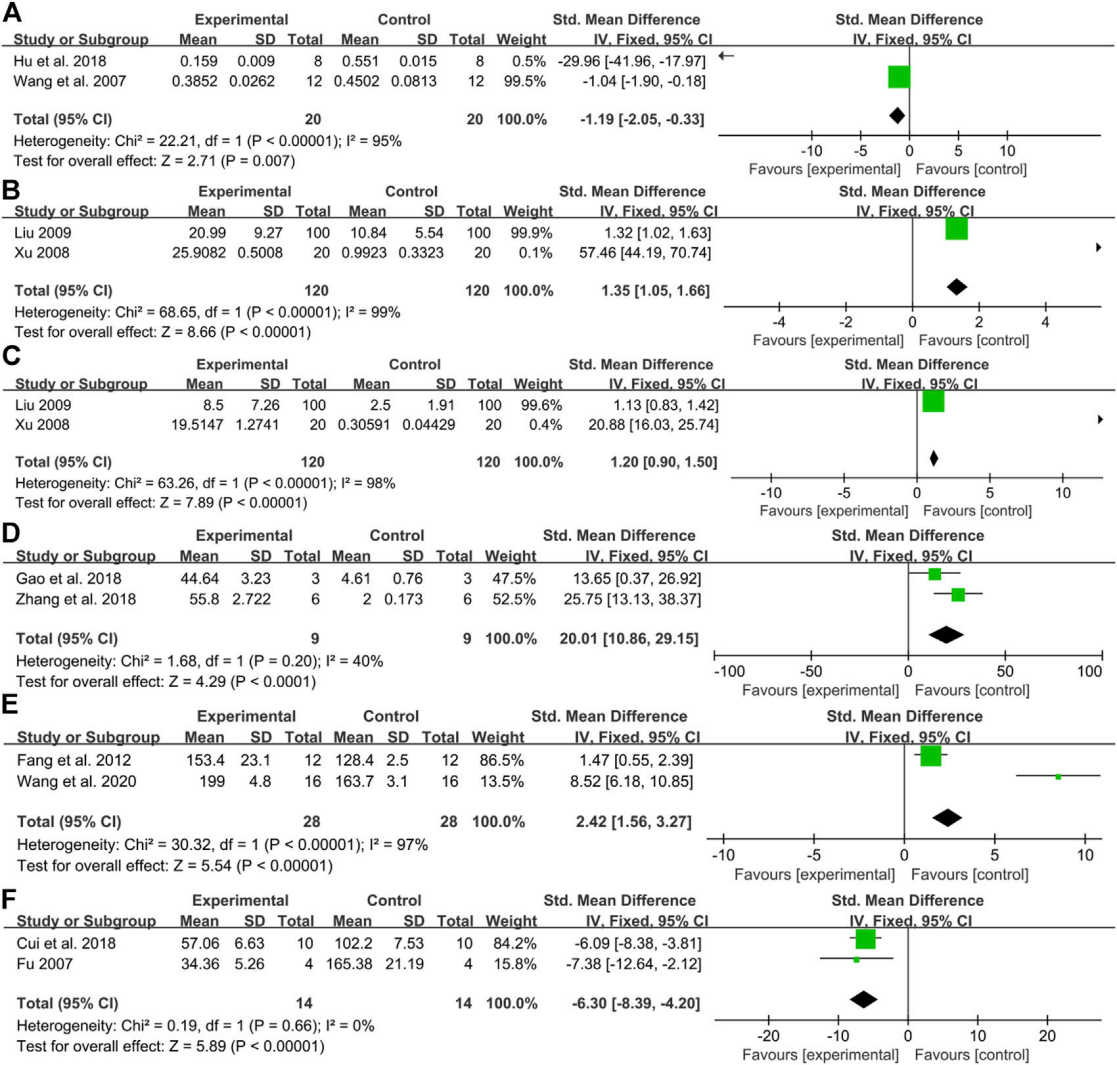
Pooled estimate of DNA damage: **(A)** cell viability, **(B)**TDNA%, **(C)** TM, **(D)** apoptosis rate, **(E)** heart rate, and **(F)** Ca^2+^ transient amplitude.

When a toxic substance acts on mammalian cells, the most obvious manifestation is a decrease in cell vitality. Therefore, cell viability is often used as target organ toxicity for rapid and mass screening of natural products. In the present study, fifteen articles measured the index of cell viability, among which ten articles revealed that aconitine signally inhibited cardiomyocyte activity in a dose-dependent manner. Comparatively, there was evidence from one article that aconitine (5–1,280 μM) had no obvious effect on myocardial cell viability compared to the control group (Liu, 2007). A little regrettably, two articles did not carry out statistical analysis (Xu, 2008; Zhou et al., 2020). The data integrity analysis from two articles manifested a noticeable decline in myocardial cell viability by aconitine administration [*n* = 40, SMD = −1.19, 95% CI (−2.05 to −0.33), *p* = 0.007; heterogeneity: *χ* ^2^ = 22.21, df = 1 (*p* < 0.00001), I^2^ = 95%] (Figure 3A) (Wang et al., 2007; Hu et al., 2018).

#### DNA Damage

As the main signal of cell apoptosis, the serious DNA damage cannot be repaired, resulting in programmed death. In our results, three studies investigated the effect of aconitine in DNA damage, including HDNA%, TDNA%, TM, TL, and OTM (Fu, 2007; Xu, 2008; Liu, 2009). It is found that HDNA% decreased gradually, while TDNA%, TM, TL, and OTM increased gradually after aconitine administration with concentration dependency. The meta-analysis of two studies (Xu, 2008; Liu, 2009) showed a significant increase in TDNA% [*n* = 240, SMD = 1.35, 95% CI (1.05 to 1.66), *p* < 0.00001; heterogeneity *χ* ^2^ = 68.65, df = 1 (*p* < 0.00001), *I* ^2^ = 99%] (Figure 3B), and a significant increase in TM [*n* = 240, SMD = 1.20, 95% CI (0.90 to 1.50), *p* < 0.00001; heterogeneity *χ* ^2^ = 63.26, df = 1 (*p* < 0.00001), *I* ^2^ = 98%] (Figure 3C).

#### Apoptosis Rate

A large number of cardiomyocyte apoptosis is also considered a crucial marker for evaluating the cardiotoxicity of compounds (Hu et al., 2018). In our study, the apoptosis rate was conducted in seven studies (Gao, 2018; Hu et al., 2018; Zhang et al., 2018; Liu et al., 2019; Li et al., 2020; Xia et al., 2021; Yang et al., 2021). The meta-analysis of two studies (Gao, 2018; Zhang et al., 2018) showed that compared with the control group, the apoptosis rate prominently surged and boomed in a concentration-dependent manner [*n* = 18, SMD = 20.01, 95% CI (10.86 to 29.15), *p* < 0.0001; heterogeneity *χ* ^2^ = 1.68, df = 1 (*p* = 0.20), *I* ^2^ = 40%] (Figure 3D).

#### Heart Rate

Most drugs that act on the cardiovascular system are known to affect the heart rate to some extent. A heart rhythm that is too fast or too slow is not conducive to maintaining normal heart function. Similarly, heart rate has been used as an indispensable and flexible indicator to evaluate the cardiotoxicity of compounds (Ye et al., 2021). It was mentioned in five studies (Fu, 2007; Wang et al., 2007; Liu, 2009; Sun et al., 2014; Zhou et al., 2020) that after aconitine was applied to animals, the beating frequency of the heart was first increased, and then, the state similar to convulsion appeared. With the passage of time, there was a gradual decline in the beating frequency, and the rhythm become even worse and more random. The heart rate of SD rats was seen in clear rising in one study (Zhang et al., 2012). Conformably, aconitine-evoked a remarkable rise in heart rate was also demonstrated in zebrafish embryo models compared with the control group in a dose-response relationship (Fang et al., 2012; Cui et al., 2018; Wang M. et al., 2020; Xia et al., 2021; Ye et al., 2021). A meta-analysis of two studies (Fang et al., 2012; Wang S. et al., 2020) for the increased heart rate in zebrafish embryos [*n* = 56, SMD = 2.42, 95% CI (1.56 to 3.27), *p* < 0.00001; heterogeneity: *χ* ^2^ = 30.32, df = 1 (*p* < 0.00001), *I* ^2^ = 97%] (Figure 3E).

#### Ca^2+^ Transients

There is considerable evidence that intracellular Ca^2+^ surge can lead to irreversible damage to cardiomyocytes through interaction with oxidative stress and the proliferation of reactive oxygen species (ROS), which can eventually trigger an apoptotic cascade of cardiomyocytes or further exacerbation of pre-existing heart disease. The Ca^2+^ modulators are therefore widely used to screen drugs for the cardiovascular system (Fu et al., 2008). Compared with the controls, six studies concluded that the administration of aconitine could increase Ca^2+^ oscillation frequency, while decreasing its oscillation amplitude resulting in cardiotoxicity via overloaded intracellular Ca^2+^ and changed cardiac electrophysiological characteristics in a concentration-dependent manner (Fu, 2007; Zhou et al., 2013; Sun et al., 2014; Yu, 2015; Cui et al., 2018; Li et al., 2018). The meta-analysis of two studies indicated that aconitine treatment repressed the oscillation amplitude of Ca^2+^ [*n* = 28, SMD = −6.03, 95% CI (−8.39 to −4.20), *p* < 0.00001; heterogeneity: *χ* ^2^ = 0.19, df = 1 (*p* = 0.66), I^2^ = 0%] (Figure 3F) (Fu, 2007; Cui et al., 2018).

So to sum it up, the main mechanisms of aconitine-induced cardiotoxicity comprised impaired cell viability, ineluctable DNA damage, excessive apoptosis, abnormal heart rate, and overloaded intracellular Ca^2+^ concentration. When the value of I^2^ is greater than 50%, subgroup analysis is needed to explore the potential source of high heterogeneity. However, many included studies lack quantitative data for integration and multi-dimensional analysis, leading to the large heterogeneity in reasonably and appropriately evaluating the potential mechanisms of aconitine-induced cardiotoxicity. The future *in vitro* and *in vivo* experiments with larger samples should be urgently performed to elucidate its multifaceted molecular mechanisms on cardiotoxicity.

### Study Quality

Through a holistic and comprehensive evaluation of the included literature, 13 studies had a quality assessment range of 2/10 to 9/10 with an average of 5.92 in Table 2. Of which, one study (Sun et al., 2014) got nine points, one study (Peng et al., 2020) got eight points and four studies (Wang M. et al., 2020; Li et al., 2020; Xia et al., 2021; Ye et al., 2021) got seven points, two studies (Cui et al., 2018; Liu et al., 2019) got six points, two studies (Fang et al., 2012; Wang, 2013) got five points, two studies (Liang et al., 1991; Zhang et al., 2012) got four points, and the rest of the study (Deng, 2010) got two points. All studies were published in peer-reviewed journals. Nine studies (Fang et al., 2012; Sun et al., 2014; Cui et al., 2018; Liu et al., 2019; Wang S. et al., 2020; Li et al., 2020; Peng et al., 2020; Xia et al., 2021; Ye et al., 2021) described control of the temperature. Eight studies (Liang et al., 1991; Fang et al., 2012; Zhang et al., 2012; Wang, 2013; Sun et al., 2014; Cui et al., 2018; Peng et al., 2020; Xia et al., 2021) were randomly assigned to treatment or control groups. Six studies (Sun et al., 2014; Cui et al., 2018; Wang M. et al., 2020; Li et al., 2020; Xia et al., 2021; Ye et al., 2021) described allocation concealment. All studies declared that the outcome assessment was blinded. The anesthetics used in the four studies (Wang, 2013; Sun et al., 2014; Liu et al., 2019; Peng et al., 2020) had no apparent intrinsic vascular protection activity. No studies used animals with associated comorbidities. One study (Deng, 2010) did not calculate the sample size in the experiment. Seven studies (Sun et al., 2014; Liu et al., 2019; Wang S. et al., 2020; Li et al., 2020; Peng et al., 2020; Xia et al., 2021; Ye et al., 2021) referred to abide by animal welfare regulations. Also, five studies (Sun et al., 2014; Wang M. et al., 2020; Li et al., 2020; Peng et al., 2020; Ye et al., 2021) contained notes on potential conflict of interests.

**TABLE 2:** Quality assessment of the experiments included in the studies.

Study (year)	1	2	3	4	5	6	7	8	9	10	Total
Liang et al. (1991)	+	-	+	NR	+	NR	-	+	NR	NR	4
Zhang et al. (2012)	+	-	+	NR	+	-	-	+	NR	NR	4
Fang et al. (2012)	+	+	+	NR	+	NR	-	+	NR	NR	5
Cui et al. (2018)	+	+	+	+	+	NR	-	+	NR	NR	6
Li et al. (2020)	+	+	-	+	+	NR	-	+	+	+	7
Sun et al. (2014)	+	+	+	+	+	+	-	+	+	+	9
Liu et al. (2019)	+	+	-	NR	+	+	-	+	+	NR	6
Ye et al. (2021)	+	+	-	+	+	NR	-	+	+	+	7
Wang et al. (2020b)	+	+	-	+	+	NR	-	+	+	+	7
Deng (2010)	+	NR	-	NR	+	-	-	-	NR	NR	2
Xia et al. (2021)	+	+	+	+	+	NR	-	+	+	NR	7
Wang (2013)	+	NR	+	NR	+	+	-	+	NR	NR	5
Peng et al. (2020)	+	+	+	NR	+	+	-	+	+	+	8

1: peer-reviewed publication; 2: statements describing control of temperature; 3: randomization to treatment group; 4: allocation concealment; 5: blinded assessment of outcome; 6: use of anesthetic had no apparent intrinsic myocardial preservation or neuroprotective effect; 7: use of animals with relevant comorbidities; 8: sample size calculation; 9: compliance with animal welfare regulations; 10: declared any potential conflict of interest; NR, not reported.

## Discussion

### Limitations

Some limitations should be taken into account when we interpret the underlying mechanisms of aconitine on cardiotoxicity through a systematic and comprehensive meta-analysis. First of all, only Chinese and English articles are searched, which may result in selection bias due to the lack of studies published in other languages. Second, each article investigating the cardiotoxicity of aconitine uses different drug administration methods, routes, doses, and times, resulting in the difference in various detection indexes and the incomparability of results. Third, the quality scores of less than five points indicate a low quality of method in included studies. Furthermore, most studies have shortcomings in randomized allocation, concealed blindness, and sample size calculation, which are core criteria for study design. Moreover, the articles do not provide primary data, leading to the failure for us to illuminate the cardiotoxicity mechanisms of aconitine based on multiple indexes by meta-analysis. Therefore, the mechanisms of aconitine on cardiotoxicity outlined in our study at different levels should be rationally viewed and explained. Overall, in the follow-up protocols on the cardiotoxicity caused by aconitine, the researchers can use a nearly identical or equivalent dose range, frequency, and route in the experimental implementation phase, so as to conduct an in-depth analysis of the mechanism with available original data.

### Implications

**FIGURE 4:**
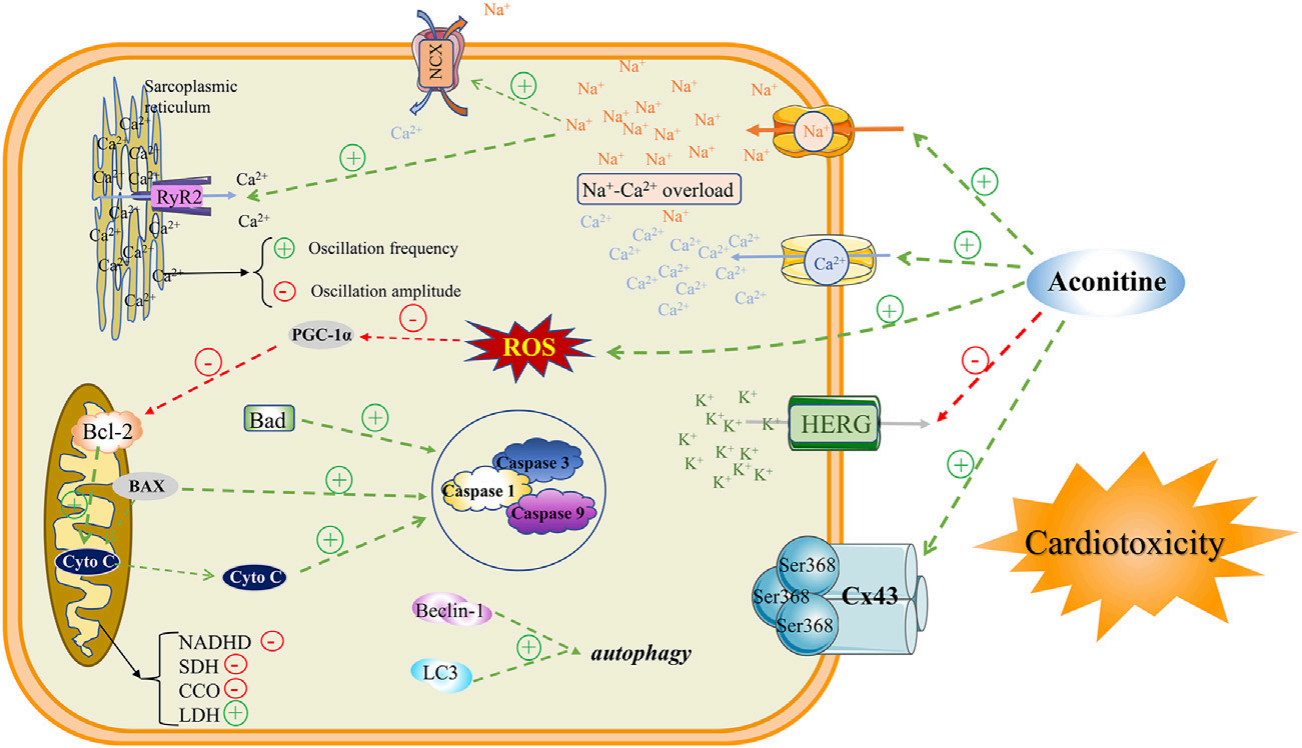
Mechanisms of aconitine-induced cardiotoxicity.

The cardiotoxicity induced by aconitine is an intricate process, involving various factors and have not been interpreted completely. Until now, the main mechanisms of cardiotoxicity studies are mainly focused on interactions with multifarious ion channels, induction of mitochondrial dysfunction, as well evoking apoptosis, and autophagy. The specific mechanism of aconitine inducing cardiotoxicity mentioned above can provide a variety of options for further exploration by scientists of the *Aconitum*. In the meanwhile, these are widely recognized indicators that should be focused on in the development and clinical application of Chinese patent medicines containing *Aconitum* medicinal plants (Figure 4).

#### Interactions With Ion Channels

Na^+^, Ca^2+^, and K^+^ channels exist widely on the surface of myocardial cell membrane and any abnormal currents generated by these ion channels will lead to cardiotoxicity. The disruption of intracellular ion homeostasis due to the interactions between aconitine and ion channels has been proverbially considered to be the key mechanism of aconitine-induced arrhythmia. Aconitine, as an agonist to bind to voltage-gated Na^+^ channels on the cell membrane, could cause abnormal activation of Na^+^ channels and memorably increase Na^+^ influx, leading to intracellular Na^+^ overload. A previous study manifested that aconitine could augment the peak of I_Na_ by irritating Na^+^ influx in a concentration-dependent manner (Wang et al., 2021). On the other hand, evidence also suggested that aconitine could sharply elevate the expression of calmodulin ryanodine receptor 2 (RyR2) and Na^+^-Ca^2+^ exchanger (NCX), which may be the main culprit of its cardiotoxicity. The RyR2 channel has been proclaimed to induce the repetitive and persistent release of Ca^2+^ from the sarcoplasmic reticulum (SR), and thereupon enter the cytoplasmic matrix through the NCX, resulting in an explosion and overload of Ca^2+^ in the cytosol of the cardiomyocytes (Fu et al., 2007; Fu et al., 2008). Although aconitine could attenuate Ca^2+^ oscillations amplitude, the increased frequency of Ca^2+^ release from the SR ultimately contributes to the upregulation in cytoplasmic Ca^2+^ (Fu, 2007). The Ca^2+^ oscillations with higher frequencies substantially dephosphorylate the gap junction protein Cx43 at the Ser368 site resulting in changes in electrophysiological characteristics of cardiomyocytes (Liu, 2009). In general, intracellular Na^+^-Ca^2+^ overload is considered to be the basic pathogenesis of aconitine-induced arrhythmias.

Judging from the currently available evidence, generous studies have announced that aconitine-induced arrhythmia is related to K^+^ channel status. As a non-selective K^+^ channel blocker, aconitine can block transient outward of K^+^ current (I_to_), ultra-rapid delayed rectifier K^+^ current (I_kur_), and fast delay rectifier outward of K^+^ current (I_Kr_). As a crucial Kv channel widely distributed in the heart, the aconitine-induced declines of Kv4.3 mRNA expression could change I_to_ current (Liu, 2007) and expressively prolong APD plateau, thus leading to arrhythmia by fluctuating cardiac electrophysiological frequency. Amazedly, a steady stream of evidence confirmed that aconitine can block the current of I_Kr_, composed of α subunit HERG and β subunit MiRP1, by intercepting the HERG channel, causing the prolonged cardiac APD and arrhythmia (Abbott et al., 1999; Wang S. et al., 2020). After aconitine treatment on cardiomyocytes, it will accommodate the expressions of ion channel-related proteins, and eventually mediate the transposition of intracellular Na^+^, Ca^2+^, and K^+^, ultimately causing arrhythmia.

#### Mitochondrial Dysfunction

After aconitine administration, the pulsation of cardiomyocytes was reduced or even disappeared with the characteristics of a shrunken cell and smaller nucleus. The results of the electron microscope showed that aconitine could induce cavities in the cytoplasm, rough endoplasmic reticulum expansion, mitochondrial swelling, and rupture, as well crest fracture. In addition to changes in mitochondrial morphology, aconitine also perturbed mitochondrial energy metabolism, evidenced by the restrained activities of reductive nicotinamide adenine dinucleotide dehydrogenase (NADHD) (Liang et al., 1991), succinate dehydrogenase (SDH), and cytochrome oxidase (CCO), while the aggrandized lactate dehydrogenase (LDH) level (Meng, 2006; Fu, 2007; Liu, 2007; Liu, 2009; Sun et al., 2014; Zhang et al., 2018; Wang S. et al., 2022). As a consequence, the inhibited oxidative phosphorylation of the tricarboxylic acid cycle tremendously boycotted and intimidated ATP synthesis (Gao et al., 2018), resulting in the impaired energy metabolism of cardiomyocytes. Meanwhile aconitine downregulated the expression of PGC-1α (Gao et al., 2018), which efficaciously impeded mitochondrial biosynthesis and ATP synthesis. In addition to ATP production and yield, mitochondrial oxidative stress signaling had also been implicated in the cardiotoxicity of aconitine, such as mitochondrial reactive oxygen species (mtROS) and 8-hydroxy-2 deoxyguanosine (8-OHDG) oxidative damage indicators. Once the cardiomyocyte’s redox state was broken up by aconitine, massive mtROS and 8-OHDG would accumulate in the cytoplasm, (Xu, 2008; Gao, 2018; Wang S. et al., 2020; Peng et al., 2020; Xia et al., 2021; Wang W. et al., 2022), further exacerbating the mitochondrial structure and function disorder. In retrospect, we reconsidered that mitochondrial energy metabolism and oxidative stress damage may be nonnegligible and momentous molecular mechanisms of aconitine-evoked cardiotoxicity.

#### Apoptosis and Autophagy

Autophagy is a process that in which a mammalian cell engulfs its own cytoplasmic proteins or organelles and wraps them into vesicles. By fusing with lysosomes to form autophagic lysosomes, the pernicious cellular wastes and products are promptly removed and cleaned up (Wang et al., 2019b). As another cell death event that determines the fate of cells, apoptosis refers to the spontaneous and orderly cell death process under the regulation of apoptosis-related genes (Wang et al., 2019a). It was reported that the expression of autophagy proteins LC3 and Beclin-1, and pro-apoptotic associated proteins caspase-3/8/9 were markedly upregulated after aconitine treatment. Simultaneously, the expression of anti-apoptotic protein Bcl-2 is visibly downregulated (Sun et al., 2014; Hu et al., 2018; Peng et al., 2020; Wang S. et al., 2022). Therefore, maintaining the dynamic balance between autophagy and apoptosis may be an effective measure to relieve and rescue the cardiotoxicity of aconitine-related traditional Chinese medicine products.

#### NLRP3 Signaling Pathway

To date, the pathway involved in aconitine-induced cardiotoxicity has also been further demonstrated experimentally. Aconitine could miraculously activate the NLRP3 signaling pathway by abnormally increasing palmitic acid levels in cardiac tissue. Conversely, aconitine treatment or silencing of the NLRP3 gene can signally confine the expressions of caspase-1, IL-18, and IL-1β (Bi et al., 2020; Peng et al., 2020). From the available evidence, excessive NLRP3 inflammasome activation may be a potential signaling pathway for aconitine-induced cardiotoxicity. However, considering NLRP3’s pivotal role in determining cell fate, its mediated oxidative stress, mitochondrial energy metabolism, as well autophagy, and apoptosis events are consequential and meaningful targets for further exploration of aconitine-induced cardiotoxicity (Wang W. et al., 2022).

### Measures to Reduce Cardiotoxicity of Aconitine

In recent years, cardiotoxic adverse events of aconite alkaloids emerge one after another, and its inevitable cardiotoxicity has critically limited the clinical use of *Aconitum* species (Zhou et al., 2021). Therefore, we have to take some effective and immediate measures to reduce its cardiotoxicity, so that it can be better used to treat a variety of clinical diseases. First, the cardiomyocyte toxicity of aconitine can be partly reduced through reasonable compatibility with some monomers, possibly via competitive occupation of toxic protein targets (Fei et al., 2015; Dong et al., 2017). Second, the toxicity of aconitine can be effectively reduced by decocting and/or boiling *Aconitum* medicinal herbs at a reasonable time and temperature. In this process, the tempestuously toxic diester-diterpenoid alkaloids are unstable and can be easily hydrolyzed into relatively less toxic monoester-diterpenoid alkaloids, and non-toxic non-esterified diterpene alkaloids (Li et al., 2022). In detail, aconitine can be hydrolyzed into benzoylaconine by removal of an acetyl group at the C-8 site via vanishing one molecule of acetic acid, followed by obtaining aconine by removal of a benzoyl group at the C-14 site via vanishing one molecule of benzoic acid at 100°C. Third, it has been widely accepted and approved that the orderly preparing process with other Chinese herbs, such as Hezi (*Terminalia chebula Retz.*) decoction, can miraculously reduce aconitine levels in *Aconitum* herbals (Li et al., 2022). For instance, the cardiotoxicity of Caowu (*Aconiti Kusnezoffii Radix*) was almost counteracted by soaking with Hezi decoction, evidenced by lessened ROS, LDH, and release of Ca^2+^, the increased mitochondrial membrane potential, the improved nuclear morphology, and the activity of Na^+^-K^+^-ATPase in H9c2 cells (Zhang et al., 2017; Han et al., 2022). In general, the cardiotoxicity caused by aconitine can be mitigated by optimal monomer compatibility, heat processing, and soaked with potential medicinal herbal materials. In addition, appropriate dosing and administration, such as local administration, are bound to reduce the cardiotoxicity of aconitine. Hopefully, the development of sustained release dosage forms of aconitine or targeted delivery systems for specific tissues and organs may be robust measures to reduce its cardiotoxicity.

## Conclusion

As a violent poison, aconitine-induced cardiotoxicity was associated with changed electrophysiological characteristics of cardiomyocytes through interactions with Na^+^, Ca^2+^, and K^+^ channels, the dysfunction of the mitochondrion, the induction of apoptosis and autophagy, as well the activation of NLRP3 related signaling pathways. Although there are many effective methods such as monomer compatibility and diversified and distinctive processing methods of traditional Chinese medicine that reduce the cardiotoxicity of aconitine still a great deal of *in vivo* and *in vitro* experiments, as well as clinical trials are urgently needed to elucidate the dynamic processes of aconitine in animals and humans. Simultaneously, in-depth investigations into the toxic mechanisms of the heart and undiscovered potential organotoxicity still need to be further probed with the help of three-dimensional microfluidic organoid models.

## Data Availability

The original contributions presented in the study are included in the article/Supplementary Material; further inquiries can be directed to the corresponding authors.
